# Delirium Assessment in Older People in Emergency Departments. A Literature Review

**DOI:** 10.3390/diseases7010014

**Published:** 2019-01-30

**Authors:** Pilar Pérez-Ros, Francisco Miguel Martínez-Arnau

**Affiliations:** 1 School of Nursing, Universidad Católica de Valencia San Vicente Mártir, Calle Espartero, 7, 46007 València, Spain; pilar.perez@ucv.es; 2 Department of Physiotheray, Universitat de València, C/Gascó Oliag 5, 46010 València, Spain

**Keywords:** psychiatric disorder, mental health, multidisciplinary approach, diagnosis, delirium, emergency departments

## Abstract

Delirium is a neuropsychiatric syndrome often manifesting in acute disease conditions, and with a greater prevalence in the older generation. Delirium in the Emergency Department (ED) is a highly prevalent problem that typically goes unnoticed by healthcare providers. The onset of a delirium episode in the ED is associated with an increase in morbidity and mortality. Because delirium is a preventable syndrome, these statistics are unacceptable. Emergency Department staff therefore should strive to perform systematic screening in order to detect delirium. Different tools have been developed for the assessment of delirium by healthcare professionals other than psychiatrists or geriatricians. Emergency Departments require delirium assessment scales of high sensitivity and specificity, suited to the characteristics of the Department, since the time available is scarce. In addition, the presence of dementia in the assessment of delirium may induce sensitivity bias. Despite the existence of numerous delirium rating scales, scales taking less than three minutes to complete are recommended. The choice of the tool depends on the characteristics of the ED. The only scale affording high sensitivity and specificity in older people with and without dementia is the Four “A”s Test (4AT); it requires no training on the part of the rater, and can be performed in under two minutes.

## 1. Characteristics of Delirium

Delirium is a neuropsychiatric syndrome frequently seen in acute disease conditions and is more common among the older generation. It is characterized by an acute onset, fluctuating course, and alterations in consciousness, orientation, memory, thinking, perception, and behavior. The development of delirium is associated with adverse outcomes [1].

Although there is consistent evidence on the impact of delirium, in daily clinical practice, it is difficult to establish a diagnosis and treatment. As a result, the disorder remains an underdiagnosed and undertreated neuropsychiatric syndrome [2].

The underlying etiology can be classified according to the presence of predisposing and triggering or triggering factors. The predisposing factors characterize those individuals that are more likely to develop delirium, while the presence of triggering factors subsequently establishes the disorder [3]. Typical predisposing factors of delirium are old age, cognitive decline, disabilities, sensory impairments, environmental changes, and multiple morbidity [4].

The most common triggering factors are acute pain, acute medical illness or infection, immobilization or the use of physical restraints, urinary retention or urinary catheterization, dehydration, environmental factors, alcohol/drug use, and psychosocial factors. Most older patients may have several triggering factors, and although only one triggering event is needed, it is more common for several co-existing factors to be implicated [1]. The most common cause of delirium in older people is the use of medications, especially routinely prescribed drugs (psychoactive agents such as benzodiazepines, narcotic analgesics, and drugs with anticholinergic effects) [5,6,7,8] (Table 1).

Delirium may be hyperactive, hypoactive, or mixed. All forms of delirium are characterized by acute changes from baseline in the ability of the patient to maintain attention and awareness, accompanied by other cognitive disturbances that develop over a short period of time (hours to days), and tend to fluctuate in severity over the course of a day [9] (Table 2).

The prevalence of delirium varies among nursing home residents, end-of-life patients, hospitalized patients, postsurgical patients, and patients admitted to the Intensive Care Unit (ICU) or Emergency Department (ED).

In older long-term care (LTC) residents, the reported prevalence varies between 15% and 70%, though the detection of hyperactive delirium is more frequent and is characterized by symptoms such as disorientation, irritability, psychomotor agitation, and visual hallucinations [10]. Hypoactive delirium in turn is dominated by symptoms of drowsiness and inactivity; as a result, it may go unnoticed, and some people experience a mix of both subtypes [11].

The prevalence of delirium in end-of-life patients approaches 85% in palliative care settings [12].

In hospitalized older patients, delirium is the most frequent complication, with an approximate prevalence of 27% [13,14].

In postsurgical patients, the disorder is conditioned to the type of surgery and the anesthesia and the procedures used. Ear, nose and throat, and general surgery pose lesser risk, with a prevalence of 12% and 13%, respectively, whereas the prevalence of delirium in patients subjected to aortic, major abdominal, or cardiac surgery is much higher (up to 29%, 50%, and 51%, respectively) [15]. 

In the ICU, the prevalence varies between 31% and 35%, being higher in patients subjected to mechanical ventilation and with comorbidities (up to 80%) [16,17]. 

Most studies of both hospitalized and institutionalized older people show a strong association between dementia and delirium, directly proportional to the degree of dementia [18]—the probability of delirium increasing by 45% in moderate dementias and 58% in severe dementias [19]. The reported prevalence of delirium among the older in ED varies between 8% and 17% [20,21].

## 2. Delirium among Older Adults in Emergency Departments

Older patients constitute the population group most commonly seen in EDs, accounting for about 50% of all care activities in such departments. This may be due to the fact that the older have greater comorbidity and chronic diseases, with a greater risk of suffering events than more younger adults. In the ED, physicians focus on the cause of the urgent problem without evaluating the older in an integral manner, despite the fact that Comprehensive Geriatric Assessment (CGA) is known to be necessary in order to assist older persons due to their bio-psycho-social complexity [22].

Delirium in the ED is a very common problem that typically goes unnoticed by healthcare providers. The lack of time to perform CGA in the ED [22] could be the reason why different studies indicate that only 16–35% of all delirium cases are identified [21]. This lack is compounded by the misconception that delirium is a benign condition and part of the aging process [23]. Emergency Department staff therefore should strive to perform systematic screening with the purpose of detecting delirium [24]. 

Delirium in the ED has a higher prevalence in older people with cognitive disorders and in patients discharged from nursing homes [25], though it is also found in people with acute disorders such as intoxications, alcohol withdrawal, or hypoglycemia [26].

## 3. Delirium Related to Emergency Department Stay

The incidence of delirium in the ED refers not only to patients admitted to the Department with symptoms of delirium, but also to the possible development of delirium during the time spent in the ED. Few studies have analyzed the incidence of delirium related to ED stay [27], despite the fact that 75% of all cases of delirium are known to be overlooked in the ED [28]. A stay of more than 12 h in the ED is an independent risk factor for the development of delirium [29]. In addition, ED stays of longer than 10 h have been associated to a more than two-fold increase in the risk of developing delirium over the following 72 h [30]. It is also necessary to screen the older at-risk during ED stay, since the symptoms also fluctuate as the hours go by [31].

## 4. Consequences of Delirium

The onset of a delirium episode in the ED is associated to increased morbidity and mortality. The duration of hospital stay increases (21 days versus 9 days in the absence of delirium), the risk of developing dementia increases within 48 months after delirium onset, and the mortality risk increases by 62%. Furthermore, affected patients suffer loss of function and increased dependence. Because delirium is a preventable syndrome, these statistics are unacceptable [32,33,34,35].

The Society for Academic Emergency Medicine Task Force has recommended delirium assessment as a key quality indicator in emergency departments [2], and investigators have considered delirium identification as a key aspect, requesting major efforts for research in this area [36].

## 5. Treatment Strategies

The first step in properly treating delirium is to identify the causal factor or series of factors, eliminate the cause if possible, and/or manage the other associated risk factors. In addition, effective communication and reorientation should be emphasized, providing reassurance for people diagnosed with delirium [37]. Prevention of risk factors or the identification of early delirium improves the prognosis [7,8].

Non-pharmacological treatment is the best option for the management of delirium if it is possible. Recent studies indicate that non-pharmacological measures not only reduce the incidence of delirium, but also prevent falls [38].

The main non-pharmacological measure is the prevention of delirium by managing or eliminating possible predisposing and triggering factors. There are non-modifiable factors such as age or sex; factors that prove difficult to manage such as depression or dementia; and easily manageable factors such as inadequate hydration. In addition, the family should be involved in the treatment, as the primary caregivers are those who know the older person and know how to take better care when calming fear, pain, or give affection and confidence. Reorientation and behavioral intervention should be attempted by maintaining continuous visual contact with the older person. Physical restraints should be avoided and a quiet environment should be favored with dim light, without noise, visits, or techniques that may affect the older even more [39].

If the non-pharmacological measures are not successful, it is necessary to use the pharmacological treatment. The pharmacological treatment is aimed at reducing the exacerbated symptomatology that is generally presented in hyperactive delirium [40]. Anticholinergics, antipsychotics, and neuroleptics may have been prescribed for the treatment of dementia and for the management of hyperactive or mixed delirium. However, this is an aspect to consider in these patients given the mortality associated with the use of these drugs [41,42]. Research in this area should be encouraged [43,44,45].

## 6. Assessment of Delirium

Following the criteria of the Statistical Manual of Mental Disorders Fourth Edition (DMS-IV), the physician (geriatrician, neurologist, or psychiatrist) makes the diagnosis. The criteria are fluctuations in consciousness (changes in the perception of the environment, with inability to maintain attention); cognitive alterations (such as alterations in language, disorientation, or memory loss); or the development of a perceptual disturbance that is not caused by previous or developing dementia [46].

Different tools have been developed for the assessment of delirium by healthcare professionals other than psychiatrists or geriatricians [47]. The two scales regarded as the gold standard alongside the clinical diagnosis using the DSM-IV-TR for analysis of the DSM scales are described below [48].

The Delirium Rating Scale Revised 98 (DRS-R-98) uses the family, letter, and nurses to obtain information about three items that allow diagnosis (time of item onset; changes in symptom severity; physical disorder) and indicates the severity of delirium based on 13 items: motor agitation, motor delay, orientation, attention, short and long term memory, visuospatial capacity, sleep/wake cycle disturbance, perceptual disturbances and hallucinations, delusions, affective lability, language, and abnormalities in the thinking process. Ratings for each item range from 0 (no impairment), 1 (mild impairment), 2 (moderate impairment), and 3 (severe impairment). The total score is obtained by adding the score of each element, and the higher the total score the greater severity in delirium there is. This tool has a sensitivity of 91–100% and a specificity of 85–100% [49]. It takes 20–30 min to obtain the score and about two h to obtain the score gathering information needed to rate the items (including family and staff interview, and reviewing of medical records). Rating of the patient is generally based on a 24-h time period. The scale can be completed by a psychiatrist, some other physician, a nurse, or a psychologist with adequate training in evaluating phenomenology. The rater must have clinical training [47] (Table 3).

The most widely used scale worldwide is the CAM (Confusion Assessment Method). Its sensitivity (94–100%) and specificity (90–95%) have been confirmed against the diagnosis of the geriatrician [50]. The CAM evaluates four cognitive elements in the short form of the instrument: (1) acute onset and fluctuating course; (2) inattention; (3) disorganized thinking; and (4) altered level of consciousness [50]. To be diagnosed with delirium, a patient must demonstrate elements 1 and 2 as well as either 3 or 4 [50]. The CAM widely used in different care environments, but is not indicated in the ED because it takes 3–5 min for cognitive testing, followed by three min for short form instrument rating and 5 min for long form instrument rating [33]. The long form comprises 10 questions and includes the following features in addition to those of the short form: disorientation; memory impairment; perceptual disturbances; psychomotor agitation and retardation; and altered sleep–waking cycle. The scale requires trained lay raters or clinicians [47] (Table 3).

Recent systematic reviews on delirium in different population groups and hospital services [48,51,52,53,54] have focused on different aspects:

Quispel-Aggenbach et al. [48] analyzed the diagnostic tests used in older people in all healthcare services. Their review examined 22 tests, of which only two—the Observational Scale of Level of Arousal (OSLA) and the Richmond Agitation and Sedation Scale (RASS)—exhibited high sensitivity and specificity in older people. Jones et al. [51] examined the best instrument for measuring the severity of delirium. Of the 42 tools studied, only 6 (the Confusion Assessment Method-Severity Score, Confusional State Examination, Delirium-O-Meter, Delirium Observation Scale, Delirium Rating Scale, and Memorial Delirium Assessment Scale) allowed adequate assessment of severity. The analysis of these authors focused on different populations and healthcare services. Oldroyd et al. [52] in turn reviewed the scales used for the assessment of delirium in vascular surgery patients. The CAM scale and DSM were the tools most widely used in these patients, without differentiating among age groups. Oh et al. [7] evaluated the diagnosis of delirium in older people in Intensive Care Units. Their review highlighted the use of two tools, due to their easy and rapid application: the 3-Minute Diagnostic Assessment (3D-CAM) and the 4A’s test (4AT). In the study published by De [53], 21 screening tools for the diagnosis of delirium in hospitalized patients were identified. The CAM scale was the tool most widely used in the hospital setting. Lastly, Bush et al. [54] analyzed the best tool for detecting delirium in palliative patients, and likewise cited the CAM scale as a tool of choice in patients of this kind.

The present review seeks to raise awareness of the importance of delirium, its risk factors and assessment tools, in order to choose the most appropriate instrument for Emergency Departments—not only for carrying out studies but also for application in daily clinical practice.

Knowledge of the most appropriate scales in Emergency Departments will undoubtedly increase the validation of scales in several languages and the conduction of studies that can be included in future systematic reviews.

## 7. Assessment of Delirium in the Emergency Department

Emergency Departments require delirium assessment scales with high sensitivity and specificity, and suited to the characteristics of the Department, since time is short; there are situations in which vital urgency prevails; and the actions to treat the patient are complex and require a large number of techniques [22].

The DSM-IV, CAM, and DRS-R-98 criteria [42] have been used as gold standards for evaluating the sensitivity and specificity of the scales used in studies among older patients in EDs. Two versions of the CAM scale were developed for departments in which a shorter version was needed: the Confusion Assessment Method for the Intensive Care Unit (CAM-ICU) and the brief Confusion Assessment Method (bCAM) [28,55].

The CAM-ICU is an adaptation of the CAM designed to be usable by clinicians in screening for delirium in the intensive care setting, particularly in application to nonverbal (intubated) patients. The CAM-ICU utilizes the CAM diagnostic algorithm. It requires trained lay raters or clinicians, and takes an estimated 2–3 min to complete [47].

Similar to the CAM-ICU, the bCAM consists of four items: altered mental state or fluctuating course, inattention, altered level of consciousness and disorganized thinking. Delirium is diagnosed if there is an alteration of the mental status or if there is a fluctuation on its course or lack of attention, and if the level of consciousness is altered or if there is a disorganized thinking. It takes under two min to complete, and requires trained lay raters or clinicians [28,47] (Table 3).

The two scales do not take as much time to complete as the CAM or DRS-R-98, but lack sufficient sensitivity. Their sensitivity and specificity levels are over 90% in critically ill patients, but they have not been validated in older patients in the ED [28,48,56] (Table 3).

Although a recent study [57] found a sensitivity of 100% and a specificity of 98%, the positive predictive value was 92%, and the negative predictive value was 100%.

Another rating scale is the Richmond Agitation Sedation Scale (RASS). This observational scale quantifies level of consciousness and takes less than 10 seconds to perform. The RASS is a 10-point scale, with four levels of anxiety or agitation (+1 to +4 [combative]); one level to denote a calm and alert state (0); and 5 levels of sedation (−1 to −5) culminating in unarousable (−5). It has high sensitivity and specificity for a score other than 0 when performed by the practitioner [58] (Table 3).

In addition, since the RASS was developed in the intensive care setting, it was modified in order to meet the characteristics of non-critical patients (the modified RASS [mRASS]). The mRASS ranges from −5 (unarousable) to +4 (combative) [59] (Table 3).

Delirium triage screen (DTS) is a brief delirium assessment tool that was developed in order to rule out delirium in a short time (<20 s) and increase delirium screening efficiency. It was originally designed to be part of nursing triage assessment in the ED and reduces the need for formal delirium assessments by 50%. The sensitivity is 98% with a specificity of 55% [58]. The assessment includes: level of consciousness by measuring the Richmond Agitation Sedation Scale (RASS) [60], and attention by spelling the word “LUNCH” backwards [61].

When the patient spells the word “LUNCH” backwards, it is recommended that the task is stopped if there is a significant pause or if the patient perseverates on a specific letter (e.g., “H-C-U...U...U”) for a significant amount of time [28] (Table 3).

Other tools that assess visuospatial alteration have been used in different studies for the screening of delirium in ED [62]. The use of these tools in the different studies is due to the fact that visuospatial deficits are present in 87% of all delirium cases [61] (Table 3).

The Clock Drawing Test (CDT) is an instrument for screening cognitive impairment in the older population. The CDT has a sensitivity of 67% and a specificity of 38%. The CDT is scored according to a three-point score system. One point is allocated for each of the following: contour, numbers, and clock hands. There are many scoring systems for the CDT; all of them seem to have excellent psychometric properties and offer similar results regarding the correlations of CDT scores to other cognitive test scores [63]. The CDT score alone is not an acceptable tool for the assessment of delirium in the older, although it shows cognitive impairment with or without delirium [64]. 

The Spatial Span Forwards (SSF) is a visual pattern recognition test based on the digit span forwards [65] that may discriminate the inattention of delirium from that of dementia [66].

The Intersecting Pentagons Test (IPT), which is part of the Mini-Mental Scale Examination (MMSE), theoretically could be useful for the screening of delirium. [67].

The Months Of The Year Backwards (MOTYB) is widely used for bedside assessment and has been found to be particularly sensitive in detecting delirium in older acute hospital inpatients. The sensitivity is 84% (95%CI 68–94), with a specificity of 90% (95%CI 82–95) [62].

The Ottawa 3 Day-Year (O3DY) [13] is a useful test in cooperative patients to quickly identify risk for cognitive dysfunction. The O3DY scale takes less than 1–2 min to perform. It is a four-item screening instrument: day and year orientation (shared by the MMSE), date orientation, spelling “world” backwards (shared by the Brief Alzheimer Screen (BAS)), testing orientation and verbal fluency. The O3DY was retrospectively derived from the Canadian Study of Health and Aging (CSHA-1), involving randomly selected sampling of Canadian adults over age 65 years. The original investigators did not prospectively assess the O3DY, nor did they evaluate the reliability or internal consistency of this instrument [68]. When compared to the CAM, the O3DY presents a sensitivity of 85.0% (95%CI 62.1–96.8) and a specificity of 57.7% (95%CI 51.8–63.6) for prevalent delirium [69] (Table 3).

The 3-Minute Diagnostic Confusion Assessment Method (3D-CAM) addresses four features: acute onset or fluctuating course; inattention; disorganized thinking; and altered level of consciousness. It is a short interview and rating scale that uses verbal responses and observations by the rater to rate the CAM diagnostic algorithm. The Estimated time required is 3 min, and training is needed. The sensitivity of this tool is 95%, with a specificity of 94% [13] (Table 3).

Lastly, the 4 “A”s Test (4AT) [66] includes four items: level of alertness [48], brief cognitive screening (the Abbreviated Mental Test-4 (AMT4) [70]), attention testing (with the MOTYB [57]) and acute variations in mental status [37].This scale is brief (<2 min), simple to administer, and does not require training. It can be performed on people with visual and hearing impairment. It does not require physical responses and it is suitable for people with agitation or drowsiness. It has high sensitivity (89.7% in non-dementia and 83.3% in dementia) and specificity (84.1% in dementia and 91.3% in non-dementia) [71] (Table 3).

A recent systematic review of the different screening tools for delirium in the older population in Emergency Departments analyzed 7 different scales. Mariz et al. [56] concluded that the CAM and CAM-ICU scales are the most widely used in Intensive Care Units and Emergency Departments, despite recent studies indicating that more appropriate specific tools are available for the latter setting [13,70]. 

Although there are recent systematic [48,56] and literature reviews [72,73] on delirium in Emergency Departments, they indicate that further research in this area should be encouraged. The delirium assessment scales used (CAM, CAM-ICU, DTS, bCAM, and others due to language validation) are diverse—the most widely studied being the CAM-ICU. Some of the included studies not only integrate Emergency Departments, but also critical care services or stay-in acute care units [48,56]. The prevalence and associated factors, such as the occurrence of delirium upon admission to the Emergency Department or during stay in the latter, are not similar to those of other hospital services such as the intensive or acute care setting.

Other tools such as the modified Confusion Assessment Method of the Emergency Department (mCAM-ED) [74] and an evaluation of single-question delirium screening tools [21] have been developed in pilot studies, though further research is needed to increase their sensitivity and specificity. 

## 8. Limitations

Narrative reviews offer limited scientific evidence in comparison with systematic reviews. However, the impossibility of complying with the requirements of a correct systematic review (articles that use a similar assessment scale, similar population and experimental conditions, a time horizon of less than 10 years [75]) led us to carry out a narrative review describing which scales are specifically used in Emergency Departments, and establishing their characteristics with the purpose of promoting research in this field. A number of studies have validated assessment scales of delirium in Emergency Departments according to language [26], or combining the assessment of delirium and dementia [76].

## 9. Conclusions

For the evaluation of delirium in the ED, we should choose the screening instrument with the greatest sensitivity and specificity—if possible, close to or equal to 100%, with inter-rater reliability, and requiring no specific equipment or unwieldy operator memorization [36]. However, a number of aspects must be taken into account when choosing the most suitable instrument:

Not all EDs are similar in terms of workload or level of complexity. The presence of a geriatrician in the ED is not always possible; in fact, not all hospitals contemplate a specialist in geriatrics. In some hospitals, beds have been reserved in the ED for older people, and these are attended by an interdisciplinary team with a geriatrician. Few hospitals have a specialized ED for older adults, and an ED specifically for older patients may not be possible in all settings. The limitations of the local healthcare economical resources are decisive in this regard [22].

Different sensitivity and specificity can be obtained depending on the person who performs the assessment. The need for training prior to use is an aspect to be taken into account in the choice of instrument. Likewise, the language of the scale can also interfere in the choice, and in this regard, it is a handicap that not all scales have been validated in all languages [39].

Despite the existence of numerous delirium rating scales, the choice of scales taking less than 3 min to complete is recommended, time-adjusted to the average rating made by practitioners in the ED [77].

It should also be noted that despite validation of the scales in ED, not all EDs assist older populations with the same characteristics. In addition, the presence of dementia in the assessment of delirium may cause sensitivity bias. There is currently a knowledge gap in the assessment of delirium in the older with and without dementia. The only scale affording high sensitivity and specificity in the older with and without dementia is the Four “A” s Test (4AT), which does not require rater training and can be completed in under 2 min [69].

## Figures and Tables

**Table 1:** Predisposing and precipitating triggering factors of delirium in older people [5,6,7,8].

Predisposing Factors	Triggering Factors
Very old age (>90 years)	Multiple drug use
Male sex	Drugs (e.g., narcotics, anxiolytics, anticholinergic agents, antidepressants, benzodiazepines and neuroleptics)
Functional dependency	Anemia
Malnutrition	Emergency visits
Depression	Autoimmune diseases
Diabetes mellitus (type 1 and 2)	Falls
Stroke	Hospital admissions
Insufficient hydration	Hypoglycemia
Epilepsy	Incontinence
Dementia	Renal failure
Parkinson	Pneumonia
Dysphagia	Pain
Hearing impairment	Infections
Visual impairment	Urinary catheter
Previous delirium	Physical restraints

**Table 2:** Clinical features of hyperactive and hypoactive delirium in older people (Adapted from [9]).

	Hyperactive	Hypoactive
Deficits in cognition	Memory impairment (for instance, patients can have an inability to remember recent events or difficulty in remembering instructions)	Memory impairment
	Disorientation (first in reference to time and then to place)	disorientation (for instance patients answering slowly to questions and without spontaneity)
Disorganized thinking	Incoherent speech and rambling or irrelevant conversation, or unclear or illogical flow of ideas	Lethargy, drowsiness, apathy
Perceptual disturbances	Illusions and misinterpretations, which arise from a false impression of an actual stimulus.	Confusion
	Visual hallucinations are the most frequent, often occurring at night	
Sleep-wake cycle disturbance	Characterized by an excessive daytime sleepiness with insomnia at night, fragmentation, and reduction of sleep or complete sleep-cycle reversal	Sometimes patients can also appear to be sedated
Disturbed psychomotor behavior	Increased motor activity	Decreased motor activity.
Others	Hyper-vigilance, restlessness, agitation, aggression, mood lability	Sluggishness or lethargy approaching stupor
	Disruptive behaviors are frequently	

**Table 3:** Characteristics of delirium testing in the Emergency Department (adapted from [48]).

Scale	Cut-off Score	Rating Time	Sensitivity % (95%CI)	Specificity % (95%CI)	Trained Rater Needed
Delirium Rating Scale Revised 98 (DRS-R-98)	>17 points	20–30 min	91–100	85–100	Y
3-Minute Diagnostic Confusion Assessment Method (3D-CAM)	1, 2 and 3 or 4 items	<3 min	95 (84–99)	94 (90–97)	Y
Brief Confusion Assessment Method (bCAM)	1, 2 and 3 or 4 items	<2 min	RA 78 (65–87) P 84 (72–92)	RA 97 (95–99) P 96 (93–97)	Y
Clock Drawing Test (CDT)	10–15 points scale	<2 min	81 (72–88)	63 (57–69)	Y
Confusion Assessment Method (CAM)	1, 2 and 3 or 4 items	10 min	94 (91–97)	84 (85–94)	Y
Confusion Assessment Method for the Intensive Care Unit (CAM-ICU)	1, 2 and 3 or 4 items	<3 min	From 95 (77–100) to 100 (80–100)	From 89 (51–100) to 93 (68–100)	Y
Delirium Triage Screen (DTS)	RASS other than 0 and LUNCH BACKWARDS > 1 error	<1 min	98 (90–100)	56 (51–61)	Y
Modified Richmond Agitation and Sedation Scale (mRASS)	Other than 0	<30 s	70 (40–85)	93 (90–96)	Y
Ottawa 3 Day-Year (3ODY)	<4	<1 min	85 (62–97)	58 (52–64)	Y
Richmond Agitation and Sedation Scale (RASS)	Other than 0	<30 s	84 (74–94)	88 (84–91)	Y
Spatial Span Forwards (SSF)	<5	<2 min	90 (84–94)	41 (35–47)	Y
The 4 “A”s Test (4AT)	4 or above	<2 min	89.7 AUC (0.927)	84.1	N
The Intersecting Pentagons Test (IPT)	>0 errors	<2 min	93 (86–96)	40 (34–46)	Y
The months of the year backwards (MOTYB)	>0 errors	<2 min	85 (78–90)	58 (52–64)	Y

RA: research assistant; P: Physician; AUC: Area under the curve; Y: Yes; N: No.
